# A Systematic Review on the Relationship Between Physical Activity and Sexual Function in Adults

**DOI:** 10.7759/cureus.51307

**Published:** 2023-12-29

**Authors:** Abdullah Almuqahwi, Haidar Alabdrabulridha, Ryhana M Aljumaiah, Aseel J Alfaifi, Muna F Alnaim, Ibrahim A Alfaifi, Noor A Alsaleh, Naeem Abdullah Alsalem, Fatimah Alsuwaylih, Atheer Ahmed Ali Almana, Fatemah Ibrahim Altaweel, Shams M Alsalman, Yaqin AlAli

**Affiliations:** 1 Family Medicine, King Faisal University, Al Ahsa, SAU; 2 Preventive Medicine, King Faisal General Hospital, Al Ahsa, SAU; 3 Internal Medicine, King Fahad General Hospital, Al Ahsa, SAU; 4 College of Pharmacy, Jazan University, Jazan, SAU; 5 Plastic and Reconstructive Surgery, King Faisal University, Hofuf, SAU; 6 Surgery, Jazan University, Jazan, SAU; 7 College of Medicine, King Faisal University, Hofuf, SAU; 8 Family Medicine, King Faisal General Hospital, Al Ahsa, SAU; 9 College of Medicine, King Faisal University, Al Ahsa, SAU; 10 Family Medicine, Al-Omran Hospital, Al Ahsa, SAU; 11 Internal Medicine, Al-Baha University, Al Baha, SAU; 12 Medicine and Surgery, Albaha University, Al Baha, SAU; 13 Internal Medicine, King Faisal University, Hofuf, SAU

**Keywords:** health-related quality of life, international index of erectile dysfunction, female sexual function index, adults, health policy, physical activity

## Abstract

Sexual function is a vital component of overall well-being and quality of life. Physical activity is known to have a profound influence on various aspects of health, but its impact on sexual function in the general population remains an under-explored area. This systematic review seeks to thoroughly examine existing evidence, aiming to establish the correlation between physical activity and sexual function in both male and female adults. We conducted a comprehensive search of electronic databases and relevant sources, following the Preferred Reporting Items for Systematic Reviews and Meta-Analyses (PRISMA) guidelines. Eligible studies were those that investigated the effects of physical activity on sexual function using the International Index of Erectile Dysfunction (IIEF-5) questionnaire and the Female Sexual Function Index (FSFI). Quality assessment was performed on the included studies, and the findings were synthesized through qualitative analysis. The review identified 12 randomized controlled trials, primarily focusing on males, with interventions ranging from home-based walking to structured exercise training. Only two studies were conducted among females. The most recommended exercise was aerobic exercise. Consistent aerobic exercise proves to be a hopeful and efficient non-drug intervention for enhancing erectile function in men. However, when considering the effects of physical exercise programs on sexual function and the quality of sexual life of females, the results present challenges in drawing clear conclusions. Health policymakers play an important role in providing guidelines and recommendations to healthcare professionals, encouraging them to prescribe exercise as a preferable alternative to pharmacological treatments for enhancing sexual functions in both men and women.

## Introduction and background

Sexual health is a cornerstone of overall well-being for individuals, couples, and families, making it a vital component of social and economic development [1,2]. The multifaceted importance of sexual health is underscored by its diverse benefits. Firstly, engaging in sexual activity contributes to improved physical health by lowering blood pressure, enhancing immune system function, and promoting better heart health [1,3]. Additionally, sexual activity plays a pivotal role in mental well-being, reducing stress and anxiety while fostering overall mental health [4]. Emotionally, it has been linked to heightened self-esteem, decreased depression, and increased libido [5]. Furthermore, the positive impact extends to improved sleep, heightened intimacy, and pain relief through natural means [6]. 

Physical activity (PA), on the other hand, has been extensively researched for its positive effects on various dimensions of health. Regular PA is known to reduce the risk of chronic diseases, improve cardiovascular health, enhance psychological well-being, and boost overall quality of life [7-9]. Given the intricate interplay between physical, psychological, and social aspects of sexual function, it is logical to consider whether PA might also play a role in promoting and maintaining sexual health. PA emerges as a crucial factor in promoting sexual health, with research highlighting its positive impact on sexual function in both men and women [10]. Studies demonstrate that even small sessions of PA can significantly enhance sexual functioning, particularly arousal, in specific subpopulations [11,12]. For women, chronic PA not only contributes to cardiovascular health but also indirectly enhances sexual satisfaction by preserving autonomic flexibility and fostering a positive body image, ultimately promoting sexual well-being [13]. A noteworthy study revealed that women engaging in up to six hours of PA per week exhibited lower sexual distress and improved clitoral artery resistance compared to their less active counterparts [14]. In men, regular aerobic exercise is linked to improved erectile function and contributes to lowering the risk of erectile dysfunction by promoting increased blood flow to the penis [15]. 

Sexual function is a fundamental component of human well-being and plays a crucial role in individuals' overall quality of life. Despite the well-established benefits of PA on various aspects of health, the relationship between PA and sexual function in both genders remains uncertain. This systematic review aims to address this gap by synthesizing the available evidence to assess the impact of PA on sexual function in both genders.

## Review

Method

Search Strategy

All studies included in the systematic review were chosen in accordance with the standardized Preferred Reporting Items for Systematic Reviews and Meta-analyses (PRISMA) guidelines [16]. To identify pertinent studies regarding the impact of PA on sexual function in the general population, a two-tiered search approach was employed. The direct search relied on four comprehensive electronic databases: MEDLINE via Ovid, SCOPUS, Google Scholar, and Web of Science. Additionally, an indirect search involved reviewing the reference lists of all relevant studies identified in the initial database search. 

For electronic databases, we used combinations of Medical Subject Headings (MeSH) or other keywords (i.e., erectile dysfunction, impotence, sexual function, sexual health, physical activity, exercises, physical exercise, physical fitness, physical endurance, aerobics, general population, adults). For a detailed search strategy, refer to Appendix 1 (Table 2) in the supplementary material. The assessment of study relevance followed a hierarchical approach, considering the title, abstract, and full manuscript.

Study Selection

The inclusion criteria comprised randomized controlled trials (RCTs) with a minimum three-week follow-up involving lifestyle modification or PA. Sexual function among males was assessed by erectile dysfunction using the International Index of Erectile Dysfunction (IIEF-5) questionnaire, with the change in IIEF score treated as a continuous variable. For studies involving female participants, the Female Sexual Function Index (FSFI) questionnaire was utilized. The IIEF-5 survey's well-established validity, clinical relevance, sensitivity to change, and widespread acceptance in research serve as the foundation for its use in our systematic review [17]. By incorporating the IIEF-5 survey into our study, we enhance the robustness and reliability of our systematic review findings. The FSFI is a widely recognized and validated questionnaire designed specifically to measure sexual function in women [18]. Exclusion criteria encompassed studies lacking randomization, a control group, or follow-up periods shorter than three weeks. Observational studies, review articles, and those not published in English or Arabic were also excluded. 

Data Extraction and Synthesis

Two researchers independently reviewed the full texts of all potentially relevant articles to determine eligibility and ensure compliance with the inclusion criteria. In instances of disagreement between the authors, the third author acted as the deciding vote. An Excel sheet (Microsoft Corporation, Redmond, Washington, USA) was used to extract the data, including the initial author's name, publication year, targeted population, country of origin, study design, participant age, sample size, intervention group size, and control group size.

The tool from the Cochrane Collaboration was employed to evaluate bias risk in the included articles [19]. Studies were classified as low-risk, unclear, or high-risk across seven domains.

Results

**Figure 1:**
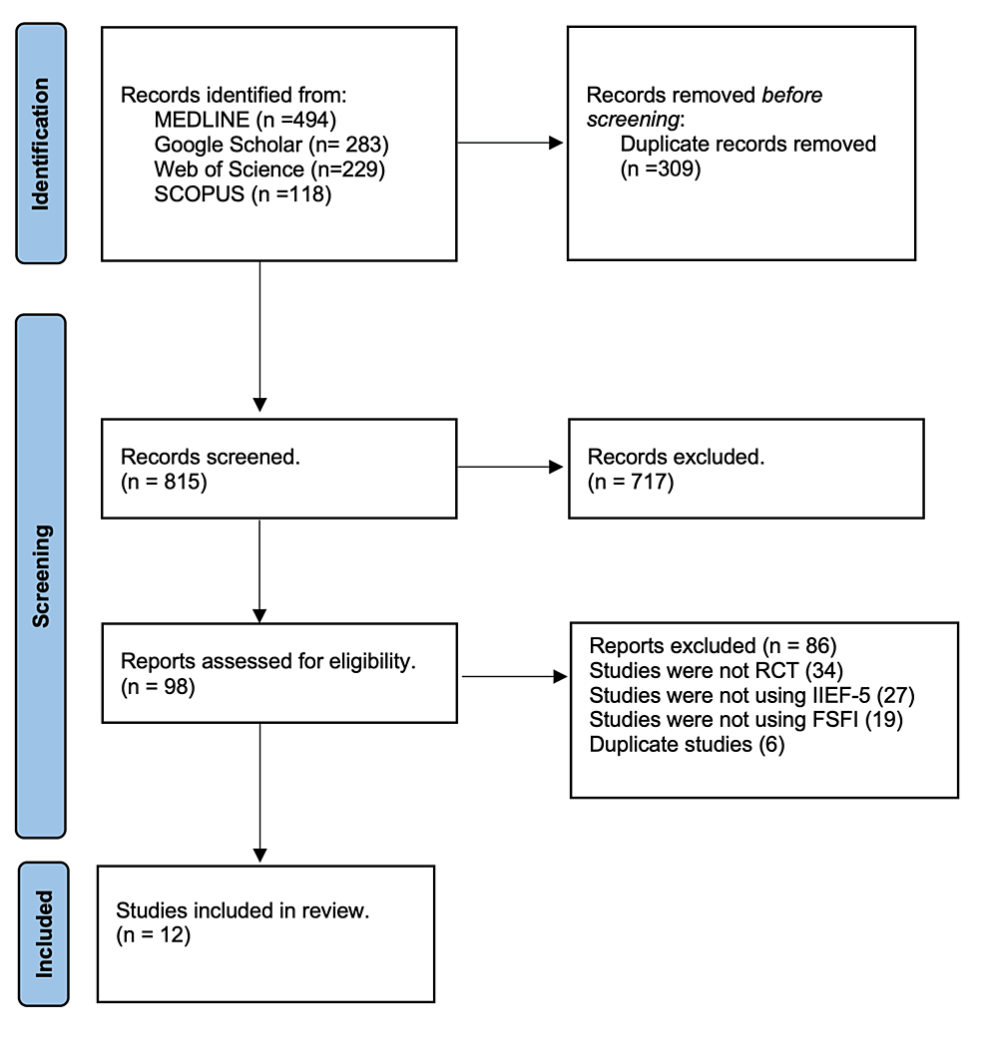
Illustrates the inclusion and exclusion of studies in this systematic review through a PRISMA flowchart. PRISMA: Preferred Reporting Items for Systematic Reviews and Meta-analyses.

The literature search identified a total of 1124 articles, and 309 duplicates were subsequently removed. After assessing the remaining 815 articles based on title and abstract, 717 were excluded. Subsequently, 98 full-text articles underwent a review for eligibility according to the inclusion criteria, resulting in the exclusion of 86 articles. In total, 12 articles were selected for qualitative analysis (Figure 1).

Characteristics of the Included Studies

Our search yielded 12 randomized controlled trials included in this systematic review [20-31], with the majority of studies (10 studies) employed an RCT design to investigate interventions for erectile dysfunction in male adults. Among the included RCTs, six were two-armed trials [20-23,30,31], one was a two-arm parallel-group design [26], one was a randomized double-blind independent group design [25], and one utilized an RCT with an assessor-blinded design [21]. These studies were conducted in diverse locations, including Brazil [20], Australia [21], Singapore [24], and Nigeria [25]. The interventions varied, incorporating approaches such as home-based walking [20], Self-help, PA and Diet using Information Technology (SHED-IT) Resources and SHED-IT Online [21], aerobic training [22], interval endurance training and general PA [23], low and high-volume exercise groups [24], exercise interval training on a bicycle ergometer [25], tadalafil treatment combined with structured PA training [26], a combination of PA, pelvic floor exercise, and psychoeducational consultations [27], and weight loss interventions including a combination of gastric bypass surgery, PA and low-energy diet [28]. The study durations varied from eight weeks [25] to 24 weeks [24], with longer-term studies extending up to two years [28]. The reported relative improvements in IIEF or IIEF-5 scores varied across studies. The outcomes across the included studies revealed significant variability in the impact of interventions on erectile function. While some studies demonstrated substantial improvements, such as the 71% decrease in erectile dysfunction observed in the home-based walking group after 30 days [20], others showed more modest gains, such as the +1.4 relative improvement in IIEF-5 score favoring the SHED-IT intervention [21]. Additionally, certain interventions, like a high-volume exercise in the Singaporean study [24], exhibited a significant mean improvement in IIEF-5 scores, highlighting the nuanced effects of PA and lifestyle interventions on erectile function outcomes (Table 1).

**Table 1:** Characteristics of the included studies.

No.	Study	Study design	Age	Gender	Study arms (N)	Arm one	Arm two	Study duration	Relative improvement
1	Begot et al. 2015 [20]	RCT	Mean age: Control group 57; Intervention group 59	M	Arm 1: Home-based walking (41). Arm 2: Control group (45)	Received usual care	Aerobic exercise; a moderate intensity	35 months	Significant 71% decrease in ED
2	Collins et al. 2013 [21]	RCT with an assessor-blinded	18–65 years	M	Arm 1: SHED-IT Resources (96) Arm, 2: SHED-IT Online, Arm 3: Control group, wait-list control group (49)	No intervention	SHED-IT Resources and SHED-IT Online, which received weight loss resource packages and, in the case of the Online group, access to a weight-loss website with e-feedback.	Six months	The reported value of +1.4 indicates a positive change in the IIEF or IIEF-5 score in favor of the intervention group.
3	Jones et al. 2014 [22]	RCT	Mean age: 59	M	Arm 1: Aerobic Training (AT) group (n = 25). Arm 2: Usual care (UC) group (n = 25).	Received usual care	Participated in five supervised walking sessions per week for 30–45 minutes per session. The intensity of the sessions ranged from 55% to 100% of VO2peak.	12-month	There were no significant between group differences in any erectile function subscale.
4	Kałkaet al. 2013 [23]	RCT	Mean age: Control group: 61.4 Intervention group: 62	M	Arm 1: Exercise group (103 subjects). Arm 2: Control group (35 subjects)	Received general health information on the need for a healthy lifestyle	Components: Interval endurance training on a cycle ergometer, indoor/outdoor general fitness exercises, and resistance training. Frequency: five days a week. Session structure: Alternating sessions of general fitness exercises and resistance training (two days a week) and interval endurance training on a cycle ergometer (three days a week). Exercise Intensity: 4-min cycling intervals with gradual intensity increase and 2-min resting periods.	Six months	Relative increase in erection quality by 15.01%
5	Khoo et al. 2013 [24]	RCT	30–60 Years	M	Arm 1: Low volume (LV) exercise group Arm 2: High volume (HV) exercise group	Prescribed exercise: Cut daily intake by ~400 kcal.; Intensity: Moderate (55–70% max heart rate). Duration: 90–150 minutes/week; Exercises: Stationary cycling, treadmill, elliptical, brisk walking, jogging, cycling, swimming.	Gradual increase in exercise duration (by 10–15 minutes/session/week) and frequency. Target exercise volume: At least 200–300 minutes/week (five to seven sessions of 30–60 minutes). Supervised exercise sessions in the research center gym initially and then at longer intervals outside the research center.	24 Weeks	The mean improvement in IIEF-5 score in men with at least moderately severe erectile dysfunction (IIEF-5 score <17) was significantly more in the HV group.
6	Lamina et al. 2009 [25]	Randomized double-blind independent group design	50-70 years	M	Arm 1: Hypertensive men in the exercise group (n = 25); Arm 2: Control group (n = 25).	Remained sedentary	The exercise group participated in a training program for eight weeks, specifically an exercise interval training program on a bicycle ergometer. The exercise sessions occurred three times per week.	Eight weeks	Pre-treatment IIEF score: 11.50±5.30. Post-treatment IIEF score: 15.14±4.92
7	Maresca et al. 2013 [26]	Two-arm parallel-group design	Mean age: 68.5	M	Arm 1: Control group (n =10 patients); Arm 2: Training group (n = 10 patients)	Received treatment with tadalafil 5 mg/day (Cialis®)	Received daily tadalafil 5 mg (Cialis®) and underwent a two-month exercise program, three 30-minute sessions per week. The program included bicycle ergometer or treadmill exercises, maintaining a target heart rate of 65% of the initial maximal oxygen consumption (VO2peak).	Two months	The IIEF score changed from 10.8 to 20.1.
8	Palm et al. 2018 [27]	RCT	Mean age: Control group 60.9 Intervention group 62.3	M	Arm 1: Control group (n = 79 patients). Arm 2: Sexual rehabilitation group (n = 75 patients)	Received usual care	Received physical exercise, pelvic floor exercise, and psychoeducational consultations for 12 weeks. The physical exercise intervention included three weekly sessions of 60 minutes each, focusing on cardiovascular training, strength exercises, and pelvic floor exercises.	12 Weeks	IIEF score at four months: The mean difference in IIEF score between the groups was 6.7 persistence of improvement at six months: The between-group mean difference in IIEF score at six months was again 6.7.
9	Reis et al. 2010 [28]	RCT	Mean age of 39.3 years	M	Arm 1: Intervention: 10 patients. Arm 2: Control: 10 patients	Received general guidance on healthy food choices and increasing physical activity from a multidisciplinary team.	It included nutritional education, a low-energy diet, and intensive behavior modification for daily physical activity, guided by a multidisciplinary team prior to gastric bypass surgery.	One year	The intervention group demonstrated no significant alterations in the IIEF-5 scores over one year period.
10	Wing et al. 2010 [29]	RCT	45–74 years	M	Arm 1: control. Arm 2: exercise total: 306 participants	Received diabetes support and education	Focused on changing diet and physical activity, with the goal of inducing a loss of 7% or more of the initial weight during the first year. Recommended physical activity was to gradually reach 175 minutes/week with moderate-intensity exercises.	One year	EF domain scores increased from 17.3 ± 7.6 at baseline to 18.6 ± 8.1 at one year.
11	Reed et al. 2014 [30]	Three by two factorial randomized controlled trial.	40-62 years old.	F	Arm 1: yoga (107). Arm 2: exercise (106). Arm 3: usual activity (142).	Received usual activity	The exercise intervention involved three weekly cardiovascular training sessions at local fitness facilities over 12 weeks. It was supervised by certified trainers. The yoga intervention included 12 weekly 90-minute classes with an emphasis on "cooling" breathing exercises, yoga poses, and guided meditation. Participants were also expected to practice yoga daily at home.	12 weeks	There was no significant difference between the yoga and usual activity groups in terms of sexual function.
12	Lorenz et al. 2013 [31]	RCT	Mean age: 32.44	F	Arm 1: Exercise before sexual activity (26). Arm 2: Exercise separate from sexual activity (26).	Participants engaged in the same duration and intensity of exercise as the experimental group but did not engage in sexual activity for at least six hours after exercising.	In the exercise group, participants exercised to a 30-minute exercise video (30-minute strength training and cardio exercise video with resistance bands. They were instructed to maintain 70–85% of their) and engaged in sexual activity immediately afterward, defined as "as soon as possible but no more than 30 minutes after the exercise video ends.	Nine-week	For the total sample of 52 women, there was a significant effect of time on sexual desire, with higher desire during the experimental exercise arm compared to pretrial and post-baseline.

Only two studies have been conducted among females to assess the effect of PA on sexual function. In the first study, led by Reed in 2014, participants were recruited from Indianapolis, IN; Oakland, CA; and Seattle, WA, focusing on women in the menopausal or postmenopausal transition aged 40-62 years old [30]. The research employed a three-by-two factorial randomized controlled trial design with three arms: Arm 1 involved yoga (107 participants), Arm 2 included PA (106 participants), and Arm 3 constituted the usual activity group (142 participants). The usual activity group served as the control and was instructed to maintain their regular PA without engaging in yoga or initiating a new PA routine. The PA intervention comprised 12 weeks of thrice-weekly cardiovascular conditioning sessions supervised by certified trainers, while the yoga intervention included both studio and home practice with an emphasis on cooling breathing exercises, specific yoga poses, and guided meditation. Among the participants, yoga showed a significant improvement in Menopause-Specific Quality of Life questionnaire (MENQOL) scores, particularly in vasomotor and sexual domains, compared to the usual activity group. In terms of sexual function, evaluated by the FSFI, there was no significant difference between the yoga and usual activity groups (P = 0.58), indicating that the yoga intervention did not lead to a notable improvement in sexual function [30]. In the other study conducted in Austin, Texas, a randomized controlled trial involved women aged 18 or older. The nine-week study included 52 participants, with the control group engaging in PA without subsequent sexual activity, and the exercise group following a 30-minute exercise video immediately followed by sexual activity [31]. The findings indicated a significant time effect on sexual desire, with higher desire observed during the experimental exercise arm compared to pre-trial and post-baseline assessments.

Risk of Bias 

**Figure 2:**
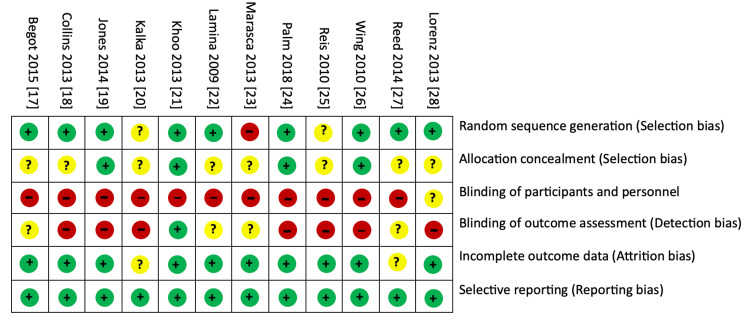
Risk of assessment of the included studies. Green (+): low risk of bias; Yellow (?): Unclear risk of bias; Red (-): high risk of bias. References: [17-28]. Note: This image is the author's own creation.

The risk of bias in the included studies was assessed and summarized in Figure 2. Across the 10 randomized trials focusing on interventions for erectile dysfunction and PA on sexual function, the overall risk of bias was moderately low. Numerous studies exhibited a low risk of bias in random sequence generation, incomplete outcome data, and selective reporting. Nonetheless, a common absence of allocation concealment descriptions was noted in the majority of trials. High-bias risks were consistently identified across all trials for performance and detection biases, primarily attributed to difficulties in blinding the treatment groups.

Discussion

The systematic review identified ten studies predominantly investigating the effects of PA on erectile dysfunction in adult males. These studies, employing diverse interventions ranging from home-based walking to structured PA training, demonstrated varying degrees of improvement in IIEF or IIEF-5 scores. Notably, the outcomes displayed a range of effects, with some interventions showcasing substantial improvements, while others exhibited more modest gains.

The nuanced impact of PA on erectile function is evident from the variability across studies. For instance, the home-based walking group showed a remarkable 71% decrease in erectile dysfunction after 30 days, emphasizing the potential benefits of a simple yet consistent PA. In contrast, the SHED-IT intervention demonstrated a more modest but still significant improvement, highlighting the importance of considering the type and intensity of PA in designing interventions. The Singaporean study, incorporating high-volume exercise, further highlights the intricate relationship between exercise intensity and its impact on erectile function. Clearly, PA has proven to be an effective, non-invasive, and non-pharmacologic intervention for reducing ED [15]. A recent meta-analysis aimed at investigating the levels of PA required to enhance the erectile function of men supports this notion. The findings of the review concluded that regular aerobic exercise indeed contributes to an improvement in the erectile function of men [15]. 

Engaging in PA has a positive impact on cardiovascular health, a factor intricately linked to erectile function [32]. Moreover, consistent aerobic exercise plays a crucial role in reducing body weight, lowering blood pressure, and enhancing glycemic control, particularly beneficial for individuals who are overweight, obese, or managing diabetes-related conditions that pose risk factors for ED [15]. By addressing these risk factors through PA, there is a potential for substantial improvement in the sexual function of men. These findings emphasize the need for tailored interventions that consider individual preferences, health status, and lifestyle factors. Additionally, the varying durations of the interventions (ranging from eight weeks to two years) suggest that long-term adherence to PA may be a crucial factor in achieving sustained improvements in erectile function. However, the findings suggest that PA does not seem to have a significant impact on prostate cancer patients. In a study conducted by Jones on cancer patients, the effectiveness of aerobic training in reducing the prevalence of ED after radical prostatectomy (RP) was investigated, comparing it to UC in a group of 50 men. Surprisingly, despite significant improvements in cardiovascular mechanisms, the study revealed that AT did not specifically improve erectile function in the short or long term after RP compared to the recommended UC. Additionally, the study raises the hypothesis that the intervention duration (six months) may be inadequate to impact erectile function. Exploratory analyses at 12 months did not reveal significant differences between aerobic training and UC. 

Despite the well-established importance of sexual health for both genders, the systematic review revealed a significant gap in research focusing on females. Only two studies were included, both exploring the relationship between PA and sexual function in women. The studies differed in their designs, one involving menopausal or postmenopausal women in a factorial randomized controlled trial and the other including adult women in a nine-week study. Lorenz (2013) [31] sought to explore the impact of PA on sexual function in women and extended the investigation to determine the optimal timing for sexual activity. The findings went beyond establishing a correlation and delved into timing-specific effects. The study concluded that there was a noteworthy influence of time on sexual desire. Specifically, women exhibited elevated levels of sexual desire during the experimental PA arm, which involved exercising immediately before sexual activity. On the other hand, Reed (2014) [30] conducted an intriguing study among peri- and postmenopausal women, aiming to investigate the impact of various interventions, including yoga, PA, and omega-3 supplementation, on menopause-related quality of life. The study aimed to assess sexual function using the FSFI. Unfortunately, the authors only reported results between the yoga and usual activity groups, omitting information on the PA group. Consequently, the study did not provide insights into whether PA potentially impacts sexual function in this population. While both studies indicated positive effects of PA on sexual function in females, the limited number of studies underscores the need for more comprehensive research in this area. Future investigations should explore the diverse aspects of female sexual function and consider factors such as age, menopausal status, and specific types of PA that may influence outcomes.

The assessment of the risk of bias across the included studies revealed moderately low overall risk, with many studies exhibiting strengths in random sequence generation, incomplete outcome data, and selective reporting. However, challenges in blinding treatment groups resulted in a consistently high risk of bias in performance and detection domains. These biases are inherent in studies involving lifestyle interventions, as participants and researchers may be aware of the assigned interventions.

From a clinical perspective, the outcomes of this review carry important implications for the management of ED. Although PA is not commonly utilized in clinical practice to enhance erectile function, the findings of this review endorse the thoughtful recommendation of supervised PA as a rational approach for men dealing with ED. Physiotherapists, who specialize in guiding and supervising PA as a tool of health promotion, may play a key role in the treatment of arterial ED [33,34]. Additionally, they should possess knowledge and competence in using PA as an intervention to treat ED [35]. The review emphasizes the need for evidence-based guidelines for clinical practice that support a PA-centered approach to improving erectile function. By understanding the complex interplay of factors influencing erectile function and overall vascular health, physiotherapists and other healthcare providers can assist patients in preventing vascular diseases and, consequently, enhance their sexual health [36]. This holistic approach offers a motivational incentive for men to make lifestyle changes, contributing to both cardiovascular health and sexual well-being [37].

The implications for female sexual health remain less clear due to the limited evidence. Healthcare providers should be aware of the potential positive effects of PA on female sexual function, but more research is needed to develop evidence-based guidelines for incorporating PA interventions into the management of female sexual health issues. Physiotherapists and other healthcare providers can contribute to this area by advocating for and participating in research that explores the impact of PA on female sexual function. 

## Conclusions

The systematic review brings attention to the potential advantages of PA in augmenting sexual function. Consistent aerobic exercise stands out as a promising and effective non-pharmacological therapy for enhancing erectile function in men. However, when considering the effects of PA programs on the sexual function and quality of sexual life of female, the results present challenges in drawing clear conclusions. Health policymakers play an important role in providing guidelines and recommendations to healthcare professionals, encouraging them to prescribe PA as a preferable alternative to pharmacological treatments for enhancing sexual functions in both men and female.

While the evidence is more robust for males, the limited data on females underscore the need for additional research in this area. Further investigations should explore the optimal types, durations, and intensities of PA for promoting sexual health in females. Moreover, research should focus on elucidating the mechanisms through which PA influence’s sexual function, considering physiological, psychological, and social factors.

## Appendices

Appendix 1 

**Table 2:** Search strategy for electronic databases.

Studies were identified by electronic searches of MEDLINE via Ovid, SCOPUS, Google Scholar, and Web of Science.	
Scopus (search conducted June 28, 2023)	(Adults OR Adult population OR Mature individuals OR Human OR individual) AND (Physical activity OR Exercise OR Lifestyle modification) AND (Sexual function OR Sexual health) AND (randomized controlled trial OR controlled clinical trial OR randomized OR clinical trial) AND (Erectile Dysfunction OR IIEF-5) AND (Female Sexual Function Index OR FSFI) (LIMIT-TO (LANGUAGE, "English" OR "Arabic"))
MEDLINE via Ovid (search conducted June 24, 2023)	#1 exp Exercise/ #2 exp Physical Fitness/ #3 exp Life Style/ #4 exp Sexual Behavior/ #5 ("Physical activity" OR Exercise.mp. OR "Lifestyle modification").mp. #6 ("Sexual function" OR "Sexual health").mp. #7 Adults/ #8 4 and 5 and 6 and 7 #9 exp Randomized Controlled Trials as Topic/ #10 exp Follow-Up Studies/ #11 ("3 weeks" OR "minimum 3 weeks").mp. #12 ("Erectile Dysfunction" OR "IIEF-5").mp. #13 ("Female Sexual Function Index" OR "FSFI").mp. #14 exp Longitudinal Studies/ #15 ("Observational studies" OR "Review articles").mp. #16 ("English" OR "Arabic").mp. #17 not (15 or 16) #18 9 and 10 and 11 and (12 or 13) and 14 and 17
Google Scholar (search conducted August 17, 2023)	"Physical activity" OR "Exercise" OR "Lifestyle modification" AND "Sexual function" OR "Sexual health" AND "Adults" OR "Adult population" AND "Randomized controlled trials" OR "Clinical trials" AND "Erectile Dysfunction" OR "IIEF-5" AND "Female Sexual Function Index" OR "FSFI" AND "Follow-up" OR "Longitudinal study" AND "English" OR "Arabic" NOT "Observational studies" NOT "Review articles"
Web of Science (search conducted August 18, 2023)	#1 (ALL=("Physical activity" OR "Exercise" OR "Lifestyle modification")) #2 (ALL=("Sexual function" OR "Sexual health")) #3 (ALL=("Adults" OR "Adult population")) #4 (ALL=("Randomized controlled trials" OR "Clinical trials")) #5 (ALL=("Erectile Dysfunction" OR "IIEF-5")) #6 (ALL=("Female Sexual Function Index" OR "FSFI")) #7 (ALL=("Follow-up" OR "Longitudinal study")) #8 (ALL=("English" OR "Arabic")) #9 (NOT ("Observational studies" OR "Review articles")) #10 (#1 AND #2 AND #3 AND #4 AND #5 AND #6 AND #7 AND #8 AND #9) #11 (ALL=("Adults" OR "Adult population" OR "Mature individuals" OR "Human" OR "Individual")) #12 (#10 AND #11)
